# Reduced NK cell IFN-γ secretion and psychological stress are independently associated with herpes zoster

**DOI:** 10.1371/journal.pone.0193299

**Published:** 2018-02-21

**Authors:** Choon Kwan Kim, Youn Mi Choi, Eunsin Bae, Mihn Sook Jue, Hyung Seok So, Eung-Soo Hwang

**Affiliations:** 1 Department of Internal Medicine, Division of Infectious Diseases, Veterans Health Service Medical Center, Seoul, Korea; 2 Department of Laboratory Medicine, Veterans Health Service Medical Center, Seoul, Korea; 3 Department of Dermatology, Veterans Health Service Medical Center, Seoul, Korea; 4 Department of Psychiatry, Veterans Health Service Medical Center, Seoul, Korea; 5 Department of Microbiology and Immunology, Seoul National University College of Medicine, and Institute of Endemic Diseases, Seoul National University Medical Research Center, Seoul, Korea; Katholieke Universiteit Leuven Rega Institute for Medical Research, BELGIUM

## Abstract

The pathogenesis of herpes zoster is closely linked to reduced varicella-zoster virus-specific cell-mediated immunity. However, little is known about the interplay between natural killer cells and psychological stress in the pathogenesis of herpes zoster. This study aimed to investigate possible associations among natural killer cells, T cells and psychological stress in herpes zoster. Interferon-gamma secretion from natural killer cell, psychological stress events, stress cognition scale scores and cytomegalovirus-specific cell-mediated immunity were compared between 44 patients with herpes zoster and 44 age- and gender-matched control subjects. A significantly lower median level of interferon-gamma secreted by natural killer cells was observed in patients with a recent diagnosis of herpes zoster than in control subjects (582.7 pg/ml vs. 1783 pg/ml; *P* = 0.004), whereas cytomegalovirus-specific cell-mediated immunity was not associated with herpes zoster. Psychological stress events and high stress cognition scale scores were significantly associated in patients with herpes zoster (*P*<0.001 and *P* = 0.037, respectively). However, reduced interferon-gamma secretion from natural killer cell and psychological stress were not associated. In conclusion, patients with a recent diagnosis of herpes zoster display reduced interferon-gamma secretion from natural killer cells and frequent previous psychological stress events compared with controls. However, reduced natural killer cell activity is not an immunological mediator between psychological stress and herpes zoster.

## Introduction

Herpes zoster (HZ) results from reactivation of the latent varicella-zoster virus (VZV) after a primary VZV infection. Several risk factors for HZ have been identified to date, such as older age, depressed cell-mediated immunity (CMI), diabetes, genetic susceptibility, trauma, recent psychological stress, female gender and European ethnicity [1]. Although the role of adaptive immunity in HZ pathogenesis has been well investigated, the role of innate immunity has not. Reduced VZV-specific CMI is the most important risk factor for HZ [2–4], with VZV-specific effector T cell activity peaking at 1–3 weeks after the onset of HZ and decreasing rapidly thereafter [5]. Because natural killer (NK) cells also play important roles in the early stage of viral infection [6], NK cell activity might affect the pathogenesis of HZ. Although NK cell activity varies with age and gender among healthy individuals, only small variations are observed over a long period of time in each individual, and individuals have been subdivided into consistently high and low groups [7]. By comparing NK cell activity between patients with a recent HZ diagnosis and control subjects without a history of HZ, we aimed to investigate the possible role of NK cells in the pathogenesis of HZ.

Although psychological stress reduces overall immune function and promotes HZ [8, 9], the interplay among psychological stress, NK cell activity and HZ has not yet been clearly elucidated. NK cell activity is reduced in response to or during psychological stress [10, 11]. Based on previous findings, we hypothesized that NK cells might serve as an immunological mediator between psychological stress and HZ pathogenesis. Psychological stress can induce reactivation and shedding of cytomegalovirus (CMV) [12], and CMV infection might be a trigger for VZV reactivation in young adults [13]. Because CMV-specific immunity may be an important variable in HZ pathogenesis and a surrogate marker of overall T cell immunity, we also compared CMV-specific CMI between patients with HZ and control subjects.

## Materials and methods

### Subjects

Study participants were prospectively enrolled at the Veterans Health Service (VHS) Medical Center in Seoul, Korea from March 2016 to September 2016. This study was approved by the Institutional Review Board of the Veterans Health Service Medical Center [file number BOHUN 2015-12-002-001]. Written informed consent was obtained from the enrolled patients with HZ and control subjects. The inclusion criteria were the following: adults >18 years of age who were clinically diagnosed with HZ by dermatologists or infectious diseases specialists within 6 months of HZ onset. Subjects were excluded if they had one or more of the following conditions: fever ≥38.3°C, suspected or confirmed infection with diseases other than HZ, a VZV IgG seronegative status, current malignancy, human immunodeficiency virus infection, pregnancy or recent use of chemotherapeutic agents or immunosuppressive drugs within the past 6 months. Control subjects without a history of HZ were individually matched with the subjects with HZ at a ratio of 1:1 for age and gender. Control subjects included patients who visited the outpatient clinic in the VHS medical center and agreed to enroll in this study. The HZ histories of control subjects were evaluated according to patient memory and medical records greater than 10 years in length (the VHS adopted electronic medical records in 2004). HZ stage was simply classified as the eruptive stage, in which the skin displayed a vesicular rash, and the healing stage, in which the skin displayed a crust or healed scar. The initial clinical manifestations were determined based on interviews of subjects with HZ or were obtained from the electronic medical records. The size of skin lesions was classified as <20 cm or ≥20 cm. Numeric pain rating scale scores (1–10) were reported by patients with HZ and classified as 1–5 or 6–10. All enrolled patients with HZ and control subjects were questioned about HZ vaccination, previous surgeries, chronic illnesses, a previous history of malignancy and body mass index. Blood tests were performed to measure leukocyte counts, the levels of C-reactive protein (CRP), blood urea nitrogen (BUN), creatinine, aspartate aminotransferase (AST), alanine aminotransferase (ALT) and serum immunoglobulin (Ig), and the CMV and VZV serologic statuses at the time of enrollment. Interferon-gamma release assays (IGRAs) for NK cells and CMV-specific T cells were performed simultaneously. The raw data of all study participants were shown in S1 File.

### NK cell IGRA

One milliliter of whole blood was collected, immediately transferred to a NK-Vue^™^ test tube (ATgen^™^, Seongnam, Korea) and incubated at 37°C for 17 h [14, 15]. The NK-Vue^™^ test tube was coated with Promocar^™^ to stimulate the NK cells. Due to the small background activities compared with the test tubes, the negative control tubes were not used, as recommended in the manufacturer’s instructions (S2 File). Supernatants from the incubated tubes were stored at -70°C. An IFN-γ ELISA was performed on the thawed supernatants according to the manufacturer’s instructions (ATgen^™^, Seongnam, Korea). The lower limit of detection for the NK cell IGRA was 40 pg/ml, and the upper limit was set to 2000 pg/ml for convenience.

### CMV-specific T cell IGRA

Three milliliters of whole blood were collected and immediately transferred to each of the following 3 CMV-Quantiferon^™^ tubes (Qiagen, Hilden, Germany): a negative control tube (nil), a positive control tube (mitogen) and a CMV antigen (Ag) tube. The antigen tube contained a variety of CMV peptides, including pp65, IE-1, IE-2, pp50 and gB. After 24 h of incubation at 37°C, the supernatants were collected and stored at -70°C. An IFN-γ ELISA was performed on the thawed supernatants according to the manufacturer’s instructions. The background IGRA value from the nil tube was subtracted from the value of CMV Ag tube, and the resulting value was used in the subsequent analysis. The upper limit for the CMV-specific T cell IGRA was set to10 IU/ml for convenience.

### Stress events and stress cognition scales

Psychological stress events were classified into more than 14 life categories, such as health, financial, residential, familial, occupational, educational, sexual, religious, marital, social, abuse, separation, recent loss, self-esteem and expression problems. Subjects were interviewed to determine whether they had experienced the abovementioned stress events within the previous 6 months. The stress cognition scale was measured using a questionnaire comprising 21 questions or statements, such as ‘I feel like crying’, ‘I have become more suspicious’, ‘I feel like breaking something’, and ‘I can’t do anything’. This questionnaire was validated by correlating the stress cognition scale score with three pre-existing measures related to stress, such as the Korean version of Symptom Checklist-90-Revised (SCL-90-R), the Korean version of the global assessment of recent stress (GARS) scale, and the perceived stress questionnaire (PSQ) [16]. Each question was weighted with scores of 0–4 points according to a Likert-style answer: ‘not at all’ (0), ‘somewhat’ (1), ‘moderately’ (2), ‘very much’ (3), and ‘absolutely’ (4). The sum of each score was used as the stress cognition scale score, with a possible range of 0 to 84 points.

### Statistical analysis

Means of continuous variables for the subjects’ characteristics and laboratory parameters were compared among groups using two-sided Student’s t-tests for normally distributed variables, and medians were compared using the Mann-Whitney U test for non-normally distributed variables, such as age, stress cognition scale scores, and CMV-specific T cell and NK cell IFN-γ secretion. Median values for NK cell IFN-γ secretion among the 3 groups were compared using the Kruskal-Wallis test and adjusted using Dunn’s multiple comparison test. The distribution of categorical variables between groups were compared using Fisher’s exact test. A logistic regression analysis of individual risk factors for herpes zoster was performed. GraphPad Prism version 7.0 (GraphPad Software, La Jolla, CA, USA), SPSS version 24 (IBM, Armonk, NY, USA) and the R program (version 3.4.3) were used for statistical analyses.

## Results

### Study population

Forty-four subjects with HZ and 44 control subjects were enrolled in the present study. The demographics of the study population are shown in Table 1. The median ages of the subjects with HZ and control subjects were 71 and 71 among the HZ and control subjects, respectively. The male-to-female ratio was high (37:7) because the study subjects were enrolled at a hospital for veterans. Patients with HZ and control subjects were not matched according to chronic illnesses or histories of malignancy. Although patients with current cancer were excluded from the present study, the frequency of patients with a history of malignancy was greater in the HZ group than in the control subjects (*P* = 0.039). Based on the assumption that HZ vaccination does not affect NK cell activity, the history of HZ vaccination was not adjusted between the patients with HZ and control subjects. Therefore, fewer patients with HZ had received the HZ vaccination (N = 6) than the control subjects (N = 16). The median time intervals from vaccination to study enrollment were 22 months (range: 10–58 months) and 12 months (range: 2–24 months) in patients with HZ and control subjects, respectively. The subjects with HZ displayed more frequent psychological stress events (*P*<0.001) and higher stress cognition scale scores (*P* = 0.037) than the control subjects. Histories of smoking and alcohol consumption; previous surgeries; the mean body mass index; and chronic illnesses, such as hypertension, diabetes, ischemic heart disease and chronic renal failure, were not different between patients with HZ and control subjects.

**Table 1:** Characteristics of patients with HZ and control subjects.

Characteristics	Number of patients		*P* values
	HZ (N = 44)	Controls (N = 44)	
**Age**	71.0 [67.0; 75.5]	71.0 [68.0; 75.5]	0.927
**Men/women**	37/7 (84.1%)	37/7 (84.1%)	1.0
**Chronic illness**	31 (70.5%)	29 (65.9%)	0.819
**History of malignancy**	11 (25.0%)	3 (6.8%)	0.039
**History of HZ vaccination**	6 (13.6%)	16 (36.4%)	0.025
**Smoking**	5 (11.4%)	2 (4.5%)	0.434
**Alcohol consumption**	15 (34.1%)	17 (38.6%)	1.0
**History of surgery**	6 (13.6%)	2 (4.5%)	0.266
**Psychological stress events**	27 (61.4%)	5 (11.4%)	<0.001
**Stress cognition scale scores**	14.0 [7.5; 18.0]	9.0 [5.0; 14.0]	0.037
**Body mass index (kg/m^2^)**	24.5±2.8	23.9±3.0	0.2616

*P* values for continuous variables were calculated using a two-sided Student’s t-test or the Mann-Whitney U test. Descriptive data are presented as the means±standard deviations (SD) for normally distributed variables and medians [interquartile ranges (IQR)] for non-normally distributed variables. Fisher’s exact test was used for categorical variables.

### Laboratory parameters

The white blood cell counts, lymphocyte counts, and the AST, ALT, CRP, and serum IgG, IgA and IgM levels were not different between the patients with HZ and control subjects (Table 2). All enrolled patients with HZ and control subjects were seropositive for CMV IgG and VZV IgG. All study subjects were CMV IgM-seronegative, and 4 of the 44 (9.1%) subjects with HZ were VZV IgM-seropositive. A higher median VZV IgG level was observed in subjects with HZ than in the control subjects (4000 mIU vs. 1883 mIU/ml, *P*<0.001), whereas the CMV IgG level was not different between the patients with HZ and control subjects.

**Table 2:** Comparison of laboratory parameters between patients with HZ and control subjects.

Parameters	Number of patients		*P* values
	HZ (N = 44)	Control (N = 44)	
**WBC (cells/μl)**	5800 [5115; 7365]	5695 [5015; 6815]	0.904
**Lymphocytes (cells/μl)**	1760 [1430; 2110]	1905 [1450; 2315]	0.374
**hs CRP (mg/l)**	0.90 [0.39; 3.20]	0.75 [0.40; 1.45]	0.332
**AST (U/l)**	25.0 [22.0; 28.5]	25.0 [21.0; 30.5]	0.799
**ALT (U/l)**	23.0 [17.0; 32.5]	21.0 [17.0; 26.5]	0.579
**Creatinine (mg/dl)**	0.95 [0.81; 1.16]	1.03 [0.82; 1.15]	0.567
**IgG (g/l)**	12.5 ± 2.8	12.4 ± 2.2	0.903
**IgA (g/l)**	2.42 [1.73; 2.96]	2.34 [1.99;2.98]	0.686
**IgM (g/l)**	0.76 [0.62; 1.07]	0.80 [0.58; 1.07]	0.851
**VZV IgG levels (mIU/l)**	4000 [2334.5; 4000.0]	1883 [1263.5; 2499.0]	<0.001

*P* values for continuous variables were calculated using a two-sided Student’s t-test or the Mann-Whitney-U test. Descriptive data are presented as the means±SD for normally distributed variables and medians (IQRs) for non-normally distributed variables. Abbreviations and normal ranges: WBC, white blood cell (4–10.0 ×10^3^ cells/μl); lymphocytes (1.5–4 ×10^3^ cells/μl); hs CRP, high-sensitivity C-reactive protein (<3.0 mg/l); AST, aspartate aminotransferase (13–33 U/l); ALT, alanine aminotransferase (8–42 U/l); creatinine (0.7–1.2 mg/dl); IgG (7–16 g/l); IgA (0.7–4.0 g/l); IgM (0.4–2.3 g/l); VZV, varicella-zoster virus.

### NK cell and CMV-specific T cell IGRAs

**Fig 1:**
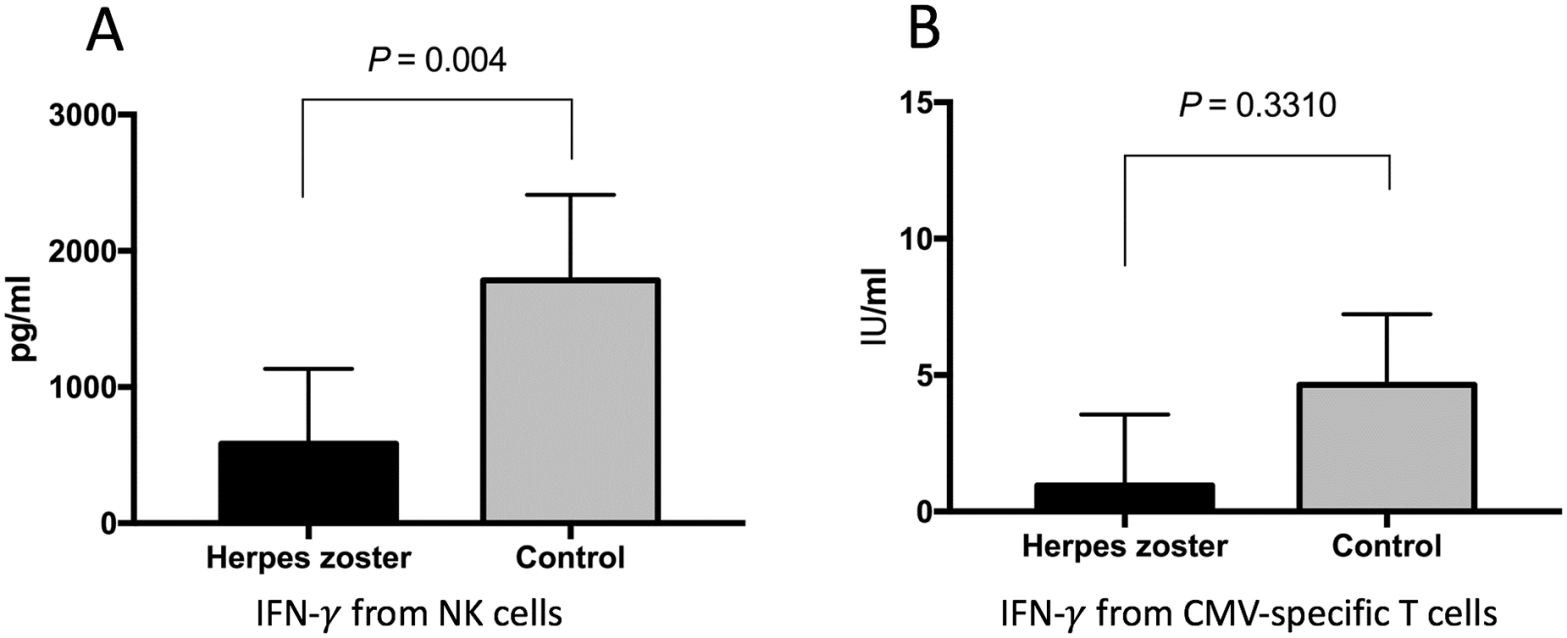
Comparison of IFN-γ levels secreted from NK cells (A) and CMV-specific T cells (B) from patients with HZ and control subjects. Median values are depicted with a 95% confidence interval bar. The *P* value was calculated using the Mann-Whitney U test.

We first compared the levels of IFN-γ secreted from NK cells between the patients (N = 44) with HZ and the control subjects (N = 44) to investigate the association between NK cell activity and HZ (Fig 1). A significantly lower median level of IFN-γ was secreted from NK cell in subjects with HZ than from those of the control subjects (582.7 pg/ml vs. 1783 pg/ml, *P* = 0.004), whereas the median level of IFN-γ secreted from CMV-specific T cells was not significantly different between the patients with HZ and control subjects (0.955 IU vs. 4.645 IU, *P* = 0.3310).

**Fig 2:**
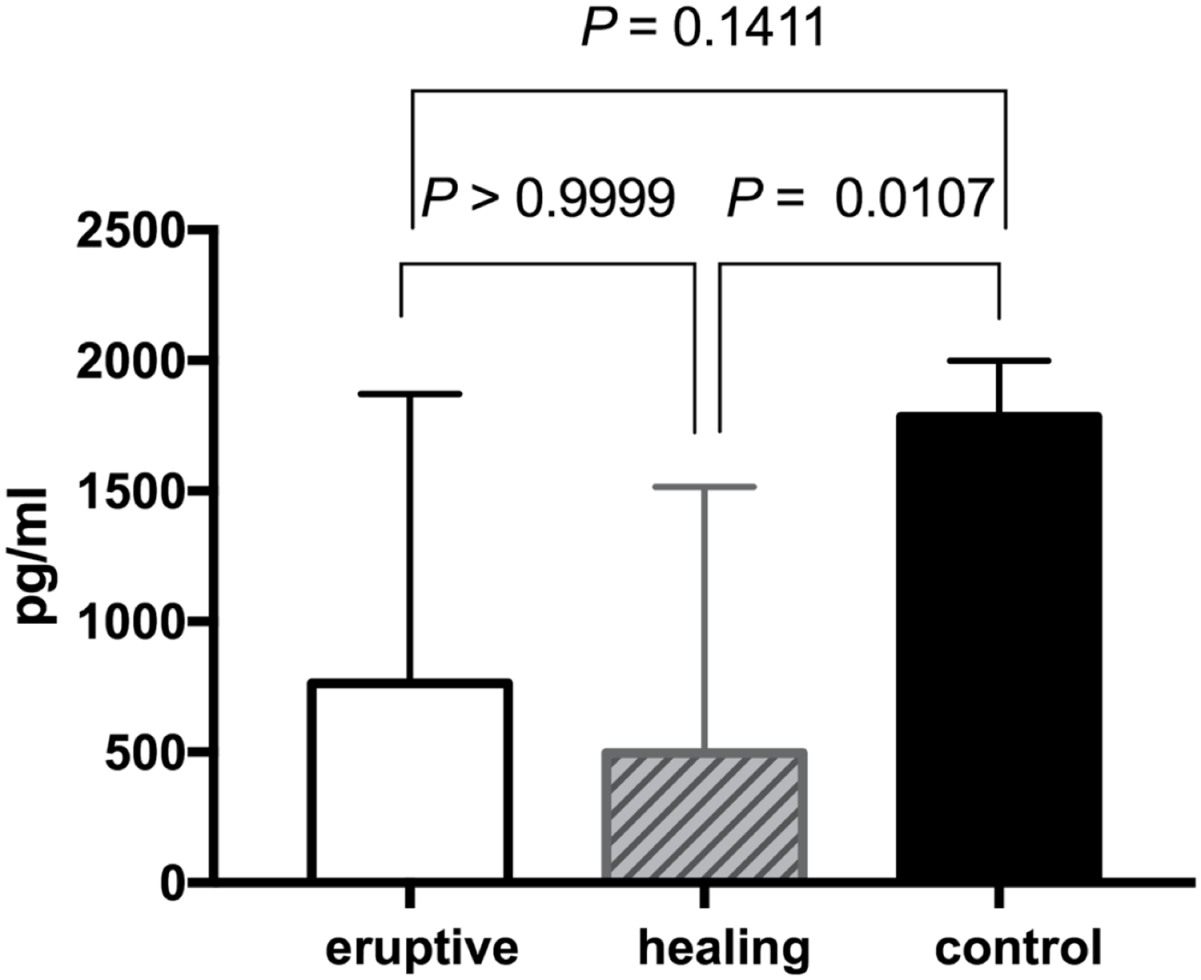
NK cell IFN-γ secretion according to HZ stage. The difference in levels of IFN-γ secreted from NK cells from subjects in the eruptive stage (N = 18), healing stage (N = 26) and control groups (N = 44) are shown. Median values are depicted with a 95% CI bar and were compared among three groups using the Kruskal-Wallis test (*P* = 0.0079). Median values were compared between two groups, and *P* values were adjusted using the Dunn’s multiple comparison test.

In a subgroup analysis (Fig 2), we analyzed the levels of IFN-γ secreted from NK cells according to the HZ stage. forty-four patients with HZ were classified into the eruptive stage group (N = 18) and the healing stage group (N = 26). The median level of IFN-γ secreted from NK cells was statistically significantly different among three groups, including the control group (*P* = 0.0079). Although the median level of IFN-γ secreted from NK cells was not different between the eruptive stage group and the healing stage group (762.5 pg/ml vs. 496 pg/ml, *P*>0.999), the levels were statistically significantly different between the healing stage group and the control group (496 pg/ml vs. 1783 pg/ml, *P* = 0.0107).

### NK cell IFN-γ secretion and psychological stress

**Fig 3:**
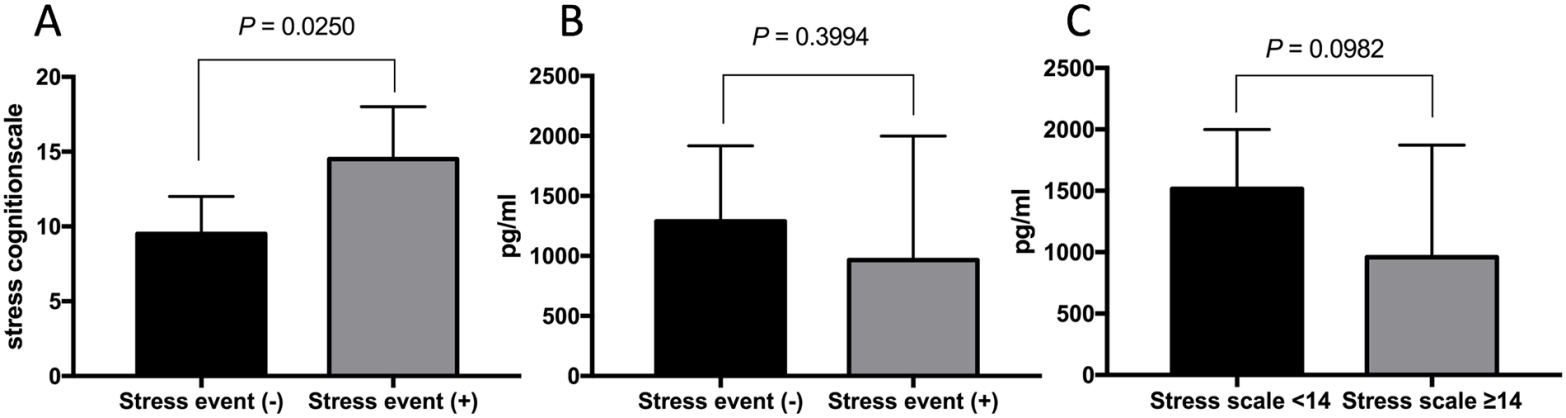
Association between psychological stress and levels of IFN-γ secretion from NK cells. The stress cognition scale scores were compared (Fig. 3A) between the subjects with psychological stress event (N = 32) and the subjects without psychological event (N = 56). The levels of IFN-γ secretion from NK cells were compared between the subjects with psychological stress event and the subjects without psychological stress event (Fig. 3B). The levels of IFN-γ secretion from NK cells were compared between the subjects (N = 49) with psychological stress cognition scale scores <14 and the subjects (N = 39) with psychological stress cognition scale scores ≥14 (Fig. 3C). The median values are depicted with 95% CIs. *P* values were calculated using the Mann-Whitney U test.

The association between NK cell IFN-γ secretion and psychological stress was analyzed in 88 enrolled subjects. The group (N = 32) that had previously experienced a psychological stress event had higher psychological stress cognition scale scores than the subjects (N = 56) who had not experienced a stress event (median 14.5 vs. 9.5, *P* = 0.0250, Fig 3A). The levels of IFN-γ secretion were compared according to psychological stress event (Fig 3B) and psychological stress scale scores (Fig 3C) which was categorized by <14 (N = 49) and ≥14 (N = 39). Neither the psychological stress event (median 1288 pg/ml vs. 965.6 pg/ml, *P* = 0.3994, Fig 3B) nor the psychological stress cognition scale score (median 1515 pg/ml vs. 959 pg/ml, *P* = 0.0982, Fig 3C) was associated with NK cell IFN-γ secretion.

### Risk factors for HZ

Multivariate logistic regression analyses were performed to identify risk factors associated with HZ compared with the control subjects. Odds ratios (ORs) and 95% CIs were estimated using logistic regression models. All variables that were identified as significant in the univariate analysis were included in the logistic regression model (Table 3, model 1). The VZV IgG levels were not included as a variable because they were tested after the occurrence of HZ. Stress cognition scale scores and NK cell IFN-γ secretion were categorized by cut-off values of 14 and 600 pg/ml, respectively, considering the small number of subjects and their distributions. We identified the effects of the independent variables associated with herpes zoster by estimating the ORs using a stepwise logistic regression model (Table 3, model 2). The following independent risk factors for HZ were identified in model 2: a history of malignancy (OR 5.403), psychological stress events (OR 20.356) and NK cell IFN-γ levels less than 600 pg/ml (OR 6.775). The variable of psychological stress cognition scale scores was dependent on the variable of psychological stress events.

**Table 3:** Logistic regression analysis of factors associated with HZ.

Risk factors	Model 1 (All variables that were significant in the univariate analysis)			Model 2 (Backward stepwise variable selection from model 1)		
	OR	95% CI	*P* values	OR	95% CI	*P* values
**History of HZ vaccination**	0.320	0.070–1.471	0.143	0.305	0.067–1.386	0.124
**History of malignancy**	5.63	1.103–28.701	0.038	5.403	1.111–26.266	0.037
**Psychological stress events**	17.87	4.431–72.099	<0.001	20.356	5.164–80.238	<0.001
**Stress cognition scale score<14**	0.49	0.147–1.655	0.252			
**NK cell IFN-γ secretion<600 pg/ml**	6.4	1.72–23.804	0.006	6.775	1.876–24.465	0.004

*P* values, OR and CI were calculated using the R program.

### Factors affecting NK cell IFN-γ secretion

The levels of IFN-γ secretion from NK cells was also compared among 44 subjects with HZ stratified according to the following factors: age, gender, chronic illness, history of malignancy, HZ vaccination, alcohol consumption, psychological stress event, stress cognition scale scores, size of the skin lesion and initial pain scale scores (Table 4). Patients with a skin lesion of more than 20 cm had lower levels of NK cell IFN-γ secretion than patients with a skin lesion less than 20 cm (*P* = 0.032). The other factors, including psychological stress events and stress cognition scale scores, were not related to NK cell IFN-γ secretion.

**Table 4:** Factors affecting IFN-γ levels in NK cells among 44 patients with HZ.

Factors	No. of patients	Median levels of IFN-γ secretion from NK cells (pg/ml)	*P* values
**Age (years)**			0.169
**<70**	19	958.5	
**≥70**	25	391.9	
**Gender**			0.759
**Male**	37	599.7	
**Female**	7	543.2	
**Chronic illness**			0.414
**Yes**	31	599.7	
**No**	13	404.8	
**History of malignancy**			0.692
**Yes**	11	599.7	
**No**	33	565.7	
**HZ vaccination**			0.352
**Yes**	6	1148.70	
**No**	38	554.45	
**Alcohol consumption**			0.431
**Yes**	16	471.05	
**No**	28	1016.80	
**Psychological stress events**			0.325
**Yes**	27	958.5	
**No**	17	391.9	
**Stress cognition scale scores**			0.348
**<14**	18	1085.45	
**≥14**	26	540.25	
**Size of skin lesions**			0.032
**<20 cm**	26	1020.25	
**≥20 cm**	18	271.05	
**Initial pain scale scores (NRS**^a^**)**			
**1–5**	22	582.7	0.887
**6–10**	22	685.6	

^a^NRS: numeric rating scale. *P* values were calculated using the Mann-Whitney U test.

### Receiver operating characteristic (ROC) curve analysis of NK cell IGRA data

**Fig 4:**
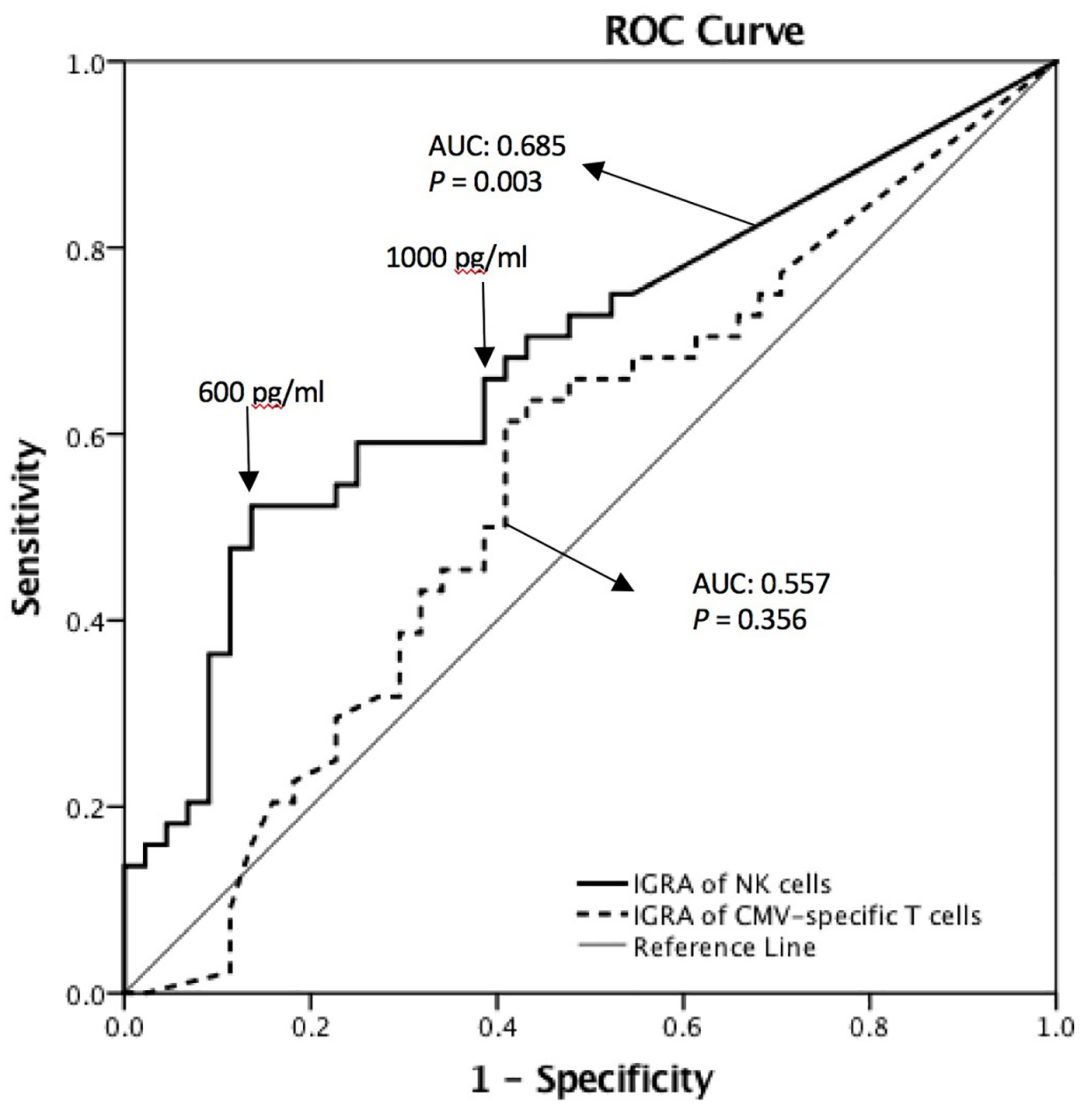
ROC curves for IGRAs using NK (continuous line) and CMV-specific T (dotted line) cells from subjects with HZ. The points corresponding to 600 pg/ml (the most distant point from the diagonal line) and 1000 pg/ml (the smallest square of the distance between the left upper corner and the curve) for the NK cell IGRA are indicated on the ROC curve. The area under the curve (AUC) and asymptotic *P* value were calculated for each test based on the assumption of nonparametric data and the null hypothesis (true area = 0.5) using SPSS software. IGRA: IFN-γ-release assay, AUC: area under the curve.

A ROC curve analysis was used to determine the utility of NK cell and CMV-specific T cell IGRAs for the diagnosis of recent HZ (Fig 4). At a cut-off value of 600 pg/ml, the sensitivity and specificity for HZ were 50% and 86.4%, respectively, and at a cut-off value of 1000 pg/ml, the sensitivity and specificity for HZ were 59.1% and 78.2%, respectively. Therefore, due to its low sensitivity and high specificity for HZ, the NK cell IGRA might be useful for differentiating between patients with recent HZ and patients without a history of HZ (area under curve: 0.685, 95% CI: 0.573–0.798, *P* = 0.003), whereas the CMV-specific T cell IGRA was not useful (area under the curve: 0.557, 95% CI: 0.436–0.679, *P* = 0.356).

## Discussion

Due to the major role of T cell-mediated adaptive immunity in HZ pathogenesis, studies of the role of NK cells in HZ have been very limited. Following elucidation of the complex mechanism of NK cell activation in the past few decades [17], studies examining the associations between the activity of NK cells and diseases were performed using recent technical advances for measuring NK cell activity [18,19]. In contrast to general concepts of NK cell activation during viral infection, we observed low levels of IFN-γ secretion from NK cell of patients with HZ compared with controls without a history of HZ. Our findings are supported by the results of two previous studies of NK cell activity in patients with HZ: NK cell cytotoxicity was reduced during HZ infection in immunocompromised hosts [20], and IFN-γ production by peripheral blood mononuclear cells (PBMCs) was decreased during the early stages of HZ infection [21]. Based on these findings, VZV activates the immune system and simultaneously uses several immune evasion mechanisms to limit NK cell activity. VZV infection was recently shown to modulate the expression of a ligand for the natural killer group 2D (NKG2D), a potent NK cell-activating receptor [22,23]. After VZV is reactivated in the dorsal ganglion and skin, the overall activity of NK cells from peripheral blood might be reduced through the modulation of NKG2D expression. However, we are unable to determine whether low levels of IFN-γ secretion from NK cell was a predisposing factor for HZ or secondary consequence of the immunomodulatory effects of VZV in the present study. Multiple sequential tests over a long period are required in the same patients with HZ to clarify the change in NK cell IFN-γ secretion after VZV reactivation.

Moreover, psychological stress events were more common and stress cognition scale scores were higher in patients with HZ than in the controls. Although psychological stress provokes the occurrence of HZ, this link is difficult to prove scientifically because HZ itself causes psychological stress [24]. We investigated both psychological stress events before HZ and the stress cognition scale scores after HZ to overcome self-reporting bias and clarify the unclear temporal relationship between psychological stress and HZ. We determined that a psychological stress event provokes HZ and influences the psychological stress cognition scale score after HZ. In addition, NK cell IFN-γ secretion was not reduced in the HZ patients with stress events compared with the HZ patients without stress events. Although this finding contradicts findings from previous studies showing that psychological stress suppresses NK cell cytotoxicity and IFN-γ production from PBMCs [25], it at least suggests that reduced NK cell activity does not mediate the association of HZ and psychological stress. According to another recent study, psychological stress simultaneously reduces perforin expression on NK cells and blood cortisol levels [26]. Although we did not compare the cortisol levels between patients with HZ and control subjects in the present study due to the diurnal variations in these levels, the association between cortisol levels and NK cell IFN-γ secretion in patients with HZ should be investigated in a future study.

Disseminated HZ involving more than 3 dermatomes can occur in immunocompromised hosts, and thus, the severity of HZ symptoms is associated with the robustness of host immune function. In fact, severe forms of varicella infection have been reported in patients with NK cell deficiency [27]. As expected, we identified a correlation between reduced NK cell IFN-γ secretion and the size of the skin lesion. Thus, NK cell immunity plays a role in the clinical manifestations of HZ. The interplay between NK cell activity and VZV-specific T cell immunity should be investigated, to determine the clinical significance of reduced levels of IFN-γ secretion from NK cell.

In the present study, IFN-γ levels in CMV-specific T cells did not differ significantly between patients with HZ and control subjects, suggesting that patients with HZ and control subjects are equally affected by immune senescence because CMV-specific CMI decreases prominently with age [28]. Although CMV-specific CMI might represent overall T cell immunity in CMV-seropositive patients, the results from the present study suggests that it does not directly reflect VZV-specific CMI in patients with HZ. Otherwise, the number of subjects in this study might be insufficient to show a statistically significant difference in the levels of IFN-γ secretion from CMV-specific T cells between patients with HZ and control subjects.

This study had three major limitations. First, we could not evaluate the interplay among NK cell activity, VZV-specific CMI and HZ because precise VZV-specific CMI measurements were difficult to perform. Although the ELISPOT (enzyme-linked immunospot) assay has been used as a standard method for measuring VZV-specific CMI measurement [29], there is no known VZV antigen or epitope available for predicting protective VZV-specific CMI. Second, subjects with HZ were clinically diagnosed based on limited serological evidence and not viral isolation or PCR, and the control subjects who participated in the present study may represent a source of bias due to incomplete memories regarding their HZ histories. Third, because the tests for NK cell activity were not performed before HZ occurred, the precise role of NK cells in HZ pathogenesis was not elucidated. In conclusion, we report that patients with a recent HZ diagnosis display reduced NK cell IFN-γ secretion activity and more frequent previous psychological stress events compared with controls. However, the reduced NK cell activity is not an immunological mediator between psychological stress and HZ pathogenesis.

## Supporting information

S1 File Case report form for 44 HZ and 44 control subjects. (XLSX)

S2 File Brief manual of NK cell IGRA. (PDF)
