# Supplementary material for: Reduced NK cell IFN-γ secretion and psychological stress are independently associated with herpes zoster
Source: PLoS One. 2018 Feb 21;13(2):e0193299. doi: 10.1371/journal.pone.0193299 (PMC5821387; doi:10.1371/journal.pone.0193299)
Supplement: S2 File — (PDF) [file pone.0193299.s002.pdf]

NK VUE
ELISA

FOR *IN VITRO* DIAGNOSTIC USE
IFN-γ ELISA ASSAY FOR MEASUREMENT OF NK CELL ACTIVITY
TO BE USED WITH NK VUE TUBE

1. INTENDED USE
The NK VUE ELISA is an IFN-γ quantitation assay for plasma samples collected and prepared with the NK VUE Tube (available separately). NK VUE is intended for *in vitro* diagnostic use, for the monitoring of the immune status of individuals. Measurement of NK cell activity could be a useful tool for assessing changes in immunosurveillance, which, in turn, could be indicative of a condition or disease where NK cell activity has been shown to be affected.

2. SUMMARY AND EXPLANATION OF NK VUE
2.1 Principle of NK VUE
NK VUE employs a proprietary stabilized immunomodulatory cytokine (Promoca) to stimulate NK cells in whole blood. After their activation, a quantitative sandwich enzyme immunoassay (ELISA) is used to determine the levels of IFN-γ secreted. To this end, an anti-IFN-γ monoclonal antibody has been pre-adsorbed on a microwell plate. Samples are pipetted into the wells and IFN-γ allowed to bind to the immobilized antibody. After washing away all unbound material, a second anti-IFN-γ monoclonal antibody conjugated to a reporter enzyme (HRP) is added to the wells. Following a final wash to remove any unbound antibody-HRP complex, the substrate solution is added to the welland color is allowed to develop.
Absorbance at 450 nm is measured, and the amount of IFN-γ released by the NK cells is finally quantitated by comparison to an IFN-γ standard curve.

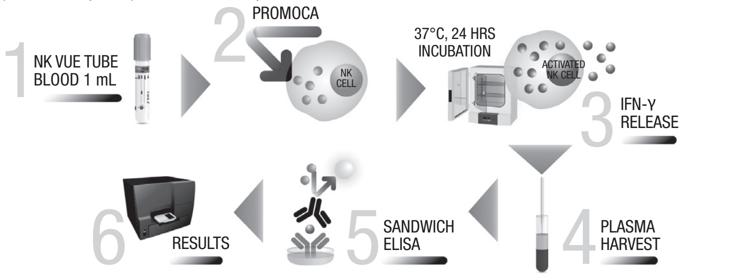

Figure 1. Principle of NK VUE

2.2 Time required for performing the NK VUE ELISA test
- Total time to manually perform the ELISA test on one full plate: ~4 hours (incubation time = 2.5 hours; hands-on time ~1.5 hours). If an automated platform is used, total time could be shortened to 3-3.5 hours.
- For each extra plate run in parallel, 15-45 minutes of hands-on time are additionally required.

3. REAGENTS AND STORAGE
3.1 Kit Components and Storage

Component	Quantity	Feature	Storage	Expiration Date	
				Unopened	Opened
**Anti-IFNγ-Coated Plate** (Microwell plate coated with a murine monoclonal antibody against human IFN-γ)	12 strips of 8 wells (equivalent to one 96-well plate)	colorless polystyrene plate	2–8 °C Protect from light	12 months	3 months
**IFN-γ Standard** (recombinant human IFN-γ)	1 vial (2 ng) (lyophilized)	white powder	2–8 °C Protect from light	12 months	3 months (≤ -20°C, resuspended)
**Diluent**	1 bottle (10 mL)	clear yellow liquid	2–8 °C Protect from light	12 months	3 months
**Biotin Conjugate (100X)** (biotin-conjugated murine monoclonal antibody against human IFN-γ)	1 vial (0.15 mL)	clear orange liquid	2–8 °C Protect from light	12 months	3 months
**Streptavidin HRP (100X)**	1 vial (0.15 mL)	clear orange liquid	2–8 °C Protect from light	12 months	3 months
**Conjugate Diluent (contains bovine serum albumin)**	1 bottle (13 mL)	clear orange liquid	2–8 °C Protect from light	12 months	3 months
**Washing Solution (20X) (contains polysorbate-20)**	1 bottle (50 mL)	colorless liquid	2–8 °C Protect from light	12 months	3 months
**TMB Solution (contains tetramethyl benzidine, TMB)**	1 bottle (12 mL)	colorless liquid	2–8 °C Protect from light	12 months	3 months
**Stop Solution (contains 1N HCL)**	1 bottle (12 mL)	colorless liquid	2–8 °C Protect from light	12 months	3 months
**High Positive Control (HPC)**	1 vial (lyophilized)	white powder	2–8 °C Protect from light	12 months	3 months
**Low Positive Control (LPC)**	1 vial (lyophilized)	white powder	2–8 °C Protect from light	12 months	3 months
**Adhesive film for microwell plate**	2 films	clear film	2–30 °C	N/A	N/A

Table 1. Kit Components and Storage Recommendations

3.2 Materials required but not provided
- Adjustable, automatic micropipettes (P200 and P1000, or similar)
- 8- or 12-channel multi-pipette, able to deliver 50 μL, 100 μL (for samples and standard), and 300 μL (for washing, optional).
- Disposable pipette tips
- Tabletop microcentrifuge (able to deliver 11,500 x g)
- Graduated cylinder (500 or 1000 mL); vortex mixer; microtube rack; microwell shaker (optional)
- Double-distilled water, or equivalent or higher grade (e.g., Milli-Q grade)
- Aspiration pump or automatic microplate washer (optional)
- Microplate reader, set to read at 450 nm (with a minimum dynamic range of 0-3.0; 0-3.5 recommended). It is highly recommended to simultaneously read at 600-650 nm (correction wavelength)

4. PRECAUTIONS
- For *in vitro* diagnostic use only.
- Use only after fully reading and understanding these guidelines.
- The TMB solution contains 3,3',5,5'-tetramethyl benzidine, a suspected carcinogen which can be harmful by ingestion, inhalation and eye or skin contact. Use eye protection, wear gloves, and handle with care.
- The Stop solution contains 1N Hydrochloric acid (HCl) which can be harmful by ingestion, inhalation, and eye or skin contact. Use eye protection, wear gloves and normal laboratory protective clothing. If the stop solution contacts skin or eyes, rinse generously with water and seek medical attention.
- Conjugate Diluent contains bovine serum albumin that can cause allergic reactions. Avoid skin contact.
- Handle human blood as if potentially infectious. Observe relevant blood handling guidelines. Wear eye protection, disposable gloves, and wash hands thoroughly after use.
- Do not use kit if any component shows signs of damage or leakage.
- When opening the lid/cap of any reagent tube/bottle or human samples, or when removing their contents, use GLP procedures to avoid microbial contamination or spraying the surroundings.
- DO NOT mix reagents/components from different kit lots.
- DO NOT use expired NK VUE ELISA components.
- When using equipment such as a plate washer or a plate reader, ensure it has been properly calibrated through a regular maintenance schedule.
- When pipetting samples or reagents, use new disposable tips and regularly calibrated pipettes.
- Discard solid waste, unused reagents and biological samples in accordance with Local, Provincial, and Federal regulations.
- Follow general laboratory safety guidelines.

5. TEST PROCEDURE
5.1 Step 1: Collection and culture of blood, and harvesting of induced plasma
- Only use plasma samples from blood that has been collected and cultured using NK VUE Tube (available separately), prepared as per the product package insert.

5.2 Step 2 – Human IFN-γ ELISA assay
Important general recommendations:
- Ensure in advance that all standards and samples can be promptly loaded onto the plate (within 15 min). This will avoid significant variations due to the time gap between loading the first and last microwell plate.
- Plasma samples, antibody-coated microwell strips and all reagents must be brought to room temperature just before use.
- Ensure that plasma samples are completely thawed and centrifuged at 11,500g for 1 min at room temperature immediately before loading them into the ELISA well.
- Microwell strips that are not required must be promptly returned to the foil pouch with desiccant, resealed, and returned to the refrigerator for storage until required.
- Washing solution (20X) must be diluted 1:19 in advance into purified water (double-distilled or higher grade). E.g., for a full plate, 50 mL of concentrated (20X) washing solution must be diluted into 950 mL of water to make a total of 1,000 mL of 1X solution. Diluted washing solution (1X) is stable at room temperature for 3 months if stored in a tightly closed bottle. If crystals appear upon storage, warm up the container at 37 °C in a water bath or incubator to re-dissolve (DO NOT use a microwave).
- For reconstituting the lyophilized IFN-γ standard, add the water and gently mix until completely dissolved (~10 min, with occasional tapping). To minimize foaming, do not mix by pipetting up and down nor vortex at high speed.
- Never leave the strip wells empty or to dry out. Always have the next solution to be pipetted prepared beforehand.

Protocol:
1. Each vial of lyophilized IFN-γ standard, High Positive Control, and Low Positive Control has to be reconstituted with 500 μL of deionized or distilled water, respectively. Mix gently to minimize frothing and ensure complete solubilization. Final IFN-γ concentrations will be 4,000 pg/mL for the IFN-γ-standard, 1,000 pg/mL for the High positive control, and 165 pg/mL for the Low positive control.
   - Of these three solutions, only 100-200 μl will be used per run. The remaining volumes of reconstituted standards and controls can be aliquoted for further use and stored frozen for up to three months (-20 °C or colder).
2. Label four 1.5 mL microcentrifuge tubes from #1 to #4 to generate the dilutions for the standard curve. Serial-dilute the standard solution as follows (see Figure 2 and Table 2): Pipet 200 μL of Diluent into tube #4, and 300 μL into tube #3 to 1, as shown in the diagram below. Make a serial dilution of the reconstituted standard (4,000 pg/mL) starting by transferring 200 μL of it into tube #4, and subsequently 100 μL from tube #4 to #3 and then 100 μL from tube #3 to #2, as shown in the diagram, and mixing well after each transfer. Do not transfer any further volume into tube #1 (0 pg/mL).

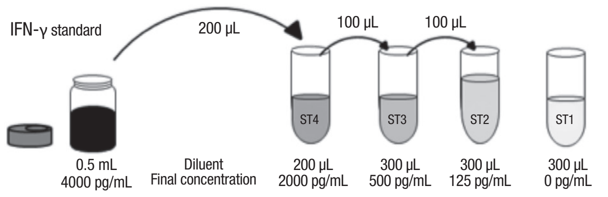

Figure 2. Preparation of standard curve dilutions

Standard dilution method (standard range = 0-2,000 pg/mL)			
ST	Diluent (μL)	Standard solution (μL) from previous dilution	Final standard concentration (pg/mL)
1	300	0	0 (blank)
2	300	100	125
3	300	100	500
4	200	200	2000

Table 2. Preparation of standard curve dilutions

3. Prepare 2 (duplicate) wells for each of the four standard curve dilutions (2,000 to 0 pg/mL), 2 wells for each high and low controls, and enough wells for all specimens to be tested. See Figure 3 below for examples of plate layout:

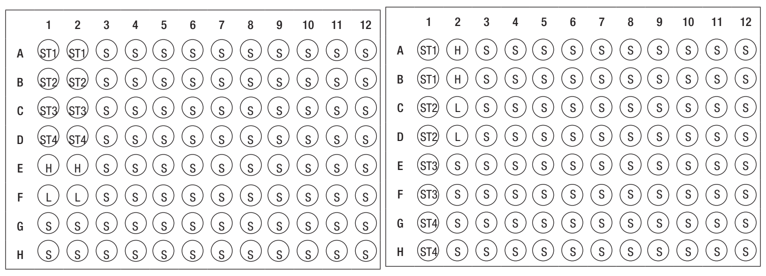

Figure 3. Suggested plate layouts
ST: Standard curve; H: High Positive Control (HPC); L: Low Positive Control (LPC); S: samples.

4. Aliquot 50 μL of diluent into each well first.
5. Add 50 μL of each dilution of the IFN-γ standard curve, 50 μL of each Low and High controls, and 50 μL of each sample to wells containing diluent. Seal plate with the adhesive film provided and lightly tap the frame to ensure proper mixing (alternatively, put for one minute in microwell plate shaker). Incubate for 1 hour at room temperature; if room temperature does not fall within the 20°C~24°C range, please refer to Table 3 for other incubation conditions.
6. Carefully remove the adhesive film from the plate and aspirate liquid from all wells. Wash 4 times by filling each well with 300 μL of Wash Buffer (1X) using a multichannel pipette, manifold dispenser, or an automated plate washer (let wash buffer sit in wells for at least 5 seconds before aspirating). Complete removal of liquid at each step is essential to good performance. After the last wash, remove any remaining wash buffer by inverting the plate and tapping vigorously on clean dry paper towels. Before aspirating the wash solution from the 4th cycle make sure Detection Solution has been prepared (see next step) to prevent drying of the wells.
7. Prepare Detection Solution by diluting Biotin Conjugate and Streptavidin HRP 1:99 into Conjugate Diluent. Example (to prepare 10 mL): first add 9.8 mL of Conjugate Diluent, then pipet 100 μL of Biotin Conjugate, and finally 100 μL of Streptavidin HRP, and mix by gentle inversion.
   - Prepare Detection Solution right before use. Detection Solution cannot be re-used nor stored.
8. Add 100 μL of Detection Solution (made in step 7) to each well. Cover plate with adhesive film. Incubate for 1hr 30min at room temperature; if room temperature does not fall within the 20°C~24°C range, please refer to Table 3.
9. Repeat the aspiration/wash cycles as in step 6 also for a total of 4 washes. After the final wash, invert and tap plate vigorously on clean absorbent paper to remove all washing solution. Before removing the final wash, have the TMB Solution prepared beforehand.
10. Add 100 μL of TMB Solution to each well. Incubate for 30 minutes at room temperature and in the dark.
    - Protect the TMB Solution at all times from strong light or sunlight.
    - For optimal reproducibility, always incubate for exactly 30 minutes.
11. Add 100 μL of Stop Solution to each well. The color in the well should change from blue to yellow.
    - For optimal reproducibility, Stop Solution should be added to wells in the same order and speed as the TMB Solution in step 9.
    - If the color in the well is green or if it does not appear uniform, gently tap the plate to ensure thorough mixing.
12. Carefully wipe clean the bottom of the plate with soft absorbent paper to remove residual humidity or foreign substances, and promptly determine the absorbance of each well using a microplate reader set to 450 nm. It is highly recommended to simultaneously read at 600-650 nm as correction wavelength..
    - Read within 5 minutes since absorbance will slowly decrease over time after adding the Stop Solution.

Remove samples from refrigerator/thaw them if frozen
↓ Spin 1 min at 11,500g
Pipet diluent, standard curve and samples (50 μL each) into pre-coated microwell strips
↓ 1st Incubation: Capture Step. Then wash 4X
Add antibody-enzyme conjugate (100 μL)
↓ 2nd Incubation: Binding Step. Then wash 4X
Add TMB solution (100 μL)
↓ 3rd Incubation: Color Development (in the dark)
Add stop solution (100 μL)
↓
Read plate at 450 nm (ELISA reader)

Figure 4 Summary of procedure

Lab Temperature	1st Incubation	2nd Incubation	3rd Incubation
**15°C~19°C**	1hr 30min	1hr 30min	30min
**20°C~24°C**	1hr	1hr 30min	30min
**25°C~37°C**	1hr	1hr	30min

Table 3. Lab temperature and incubation times

6. CALCULATION AND RESULT ANALYSIS
All data processing and calculations can be carried out using software packages available with microwell plate readers, standard spreadsheets (e.g., MS Excel) or common statistical software (e.g., GraphPad, Sigma Plot).
- Subtract the correction wavelength (600-650 nm) readings from all 450 nm readings.
- Calculate the average values of the corrected 450 nm absorbance readings corresponding to all samples (standard curve and plasma samples), and also the variation between duplicates. It is common pratice to discard a data pair if CV% is >20% (sample should be re-tested), except for low concentration samples where significantly higher CV% could be tolerated. Only the average values of the replicates will be used for subsequent analysis.
- There is no need to substract the blank from the sample readings; it is recommended to use the blank's reading as a data point in the standard curve.

6.1 Standard curve calculation
Perform linear regression analysis on the standard curve. The X axis shows the concentration of standard solution, in pg/mL, and the Y axis the (corrected) absorbance at 450 nm. The correlation coefficient (R^2) of the linear regression of the standard curve must be ≥0.98. A numerical example of the standard curve calculation is shown below (see Table 4).

Data (Table 4) and curve (Figure 5) in the example are intended for illustration only, and cannot be used to calculate results from other assays.

EXAMPLE:

Concentration (pg/mL)		Absorbance (450nm) (corrected, not blanked)			
		Duplicates		CV%	Average
**Standard 1**	0 pg/mL	0.050	0.060	9.1	0.055
**Standard 2**	125 pg/mL	0.200	0.197	0.76	0.199
**Standard 3**	500 pg/mL	0.659	0.676	1.3	0.668
**Standard 4**	2000 pg/mL	2.366	2.418	1.1	2.392

Table 4. Example of data for standard curve

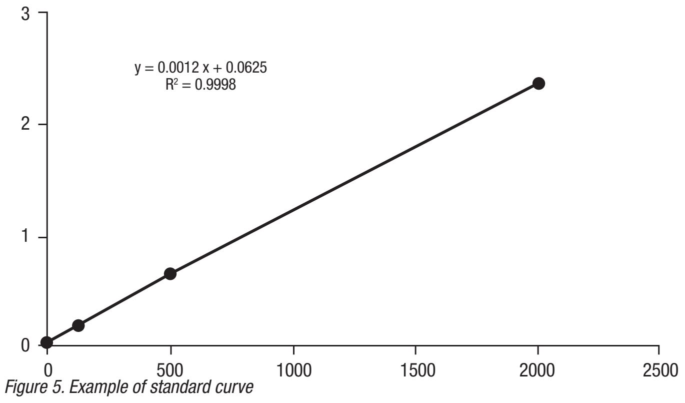

Figure 5. Example of standard curve

6.2 Quality Control
The accuracy of test results depends on the generation of an accurate standard curve. Therefore, results derived from the standards must be consistent before test sample results can be interpreted.
For the ELISA to be valid:
❶ Raw absorbance value (not blanked) of Standard 1 must be ≤ 0.15
❷ Raw absorbance value (not blanked) of Standard 3 must be ≥ 0.4
❸ Raw absorbance value (not blanked) of Standard 4 must be ≥ 1.7
❹ The correlation coefficient (R^2) of the linear regression of the standard curve must be ≥ 0.98

If these conditions are not satisfied, the test results are not valid and a new test must be conducted.

Note: After reconstitution, high and low positive controls (HPC, LPC) contain recombinant hIFN-γ at a concentration of 1,000 pg/mL and 165 pg/mL, respectively. Experimentally-determined lot-specific concentrations are indicated in the corresponding certificate of analysis included in the box.

6.3 Calculation of Sample Concentration
Calculate the concentration of the samples using the average of the absorbance values and the equation generated by linear regression analysis (y=ax+b). For instance, if the latter is y=0.0012x+0.0625 (as shown in the example), and the sample average absorbance is 1.265, then the sample concentration is calculated as follows:

Sample concentration (pg/mL) = (average absorbance of the sample – b) / a
Sample concentration (pg/mL) = (1.265-0.0625) / 0.0012 = 1002.08 (pg/mL)

Tip: if a sample shows an IFN-γ concentration >2,000 pg/mL, and there is a need to determine its precise concentration, dilute the plasma sample 1:10 with Diluent and re-assay by ELISA.

7. LIMITATIONS
Results will not be reliable if:
❶ Blood collection and culture were not properly conducted as per the package insert procedure described for the NK VUE Tube
❷ The procedure described in this package insert to carry out the ELISA test was not properly followed.

8. PERFORMANCE CHARACTERISTICS
Lower Limit of Detection: 40 pg/mL

Assay Range: 40-2000 pg/mL
Standard curve points are 2000, 500, 125, and 0 pg/mL of IFN-γ.

Precision: Inter-assay precision was evaluated as inter-session, inter-operator, inter-laboratory, inter-day and inter-lot. CV values obtained were all below 10%.

Specificity: This ELISA test is specific for human IFN-γ. No significant cross-reactivity was found against human IL1α, IL1β, IL2, IL3, IL4, IL6, IL8, IL10, IL12(p40), IL15, GM-CSF, SCF, TNFα, G-CSF, LT-α (TNF-β), VEGF165, Lymphotactin/XCL1, MIP-1a/CCL3, MCP-1/CCL2, and mouse IL1β, TNFα, IL2, and IL15. These proteins did not interfere with the IFN-γ ELISA assay of plasma containing 500 pg/mL of IFN-γ. Human albumin, bilirubin, heparin, glucose and triglycerides did not interfere with the IFN-γ ELISA assay of plasma containing 150, 300 or 500 pg/mL of IFN-γ.

Calibration: The standards in this ELISA have been calibrated to the NIAID recombinant IFN-γ standard lot Gxg01-902-535. One (1) pg of IFN-γ Standard = 0.018 NIAID units.

9. TROUBLESHOOTING

Problem	Possible cause	Solution
**Low overall sample readings**	The NK VUE tube was left for too long at room temperature before blood collection causing Promoca inactivation	Repeat blood collection using a new NK VUE tube.
	The NK VUE tube was not placed into the 37°C incubator within 15 minutes after blood collection	
**Non-specific color development / High background**	Incomplete/improper washing of the plate	Repeat ELISA, washing with 300 μL of washing solution per well, for the suggested number of times. A soak time of 5 seconds minimum should be used. Eliminate residual wash buffer by inverting plate and vigorously tapping onto absorbent paper.
	Adhesive film improperly used or sealed.	Re-using the adhesive film can cause sample cross-contamination (droplets can adhere to the inner surface). Evaporation can occur due to improper sealing.
	Cross-contamination of ELISA well	Pipet samples carefully into microwell strips to minimize risk.
	Expired components/kit	Ensure kit is used within the expiry date. Frozen samples should not be re-used, discard after thawing once.
	Substrate solution (TMB) is contaminated	Discard if solution appears blue before adding it to the wells.
**Low optical density reading for standard**	Standard dilution error	Ensure dilutions of the kit standard are prepared correctly as per the package insert.
	Incubation temperature too low or Incubation time too short	Verify incubation temperature-time ratio corresponds to Table 3. The substrate solution (TMB) must be incubated for exactly 30 minutes.
	Temperature of the solutions too low	Bring solutions to room temperature before use.
	Incorrect plate reader filter used	Plate should be read at 450 nm.
	Kit /components have expired	Ensure kit is used within the expiry date.
**Non-linear standard curve. High variability of the replicates**	Incomplete/improper washing of the plate	Repeat ELISA washing with 300 μL of washing solution per well, for the suggested number of times. A soak time of 5 seconds minimum should be used. Invert plate and tap onto absorbent paper to eliminate residual wash buffer.
	Standard dilution error	Ensure dilutions of the Kit standard are prepared as per the Package insert.
	Pipetting error	Ensure pipettes are calibrated and used according to manufacturer's instructions.
	Poor mixing	Mix reagents thoroughly prior to their use or addition to the plate.

10. TECHNICAL SERVICE
For customer technical service please contact:

Manufacturer : ATGen
SNUH HIP 4F and 6F,
172 Dolma-ro, Seongnam-si,
Gyeonggi-do 13605,
REPUBLIC OF KOREA (84141)
Tel.: 82-31-603-9212
Fax: 82-31-8017-8124
www.nkvue.com

Doc. Date: March 15, 2017
